# Novel Compound Heterozygous Mutations in TTI2 Cause Syndromic Intellectual Disability in a Chinese Family

**DOI:** 10.3389/fgene.2019.01060

**Published:** 2019-10-29

**Authors:** Rongrong Wang, Shirui Han, Hongyan Liu, Amjad Khan, Habulieti Xiaerbati, Xue Yu, Jia Huang, Xue Zhang

**Affiliations:** ^1^McKusick-Zhang Center for Genetic Medicine, State Key Laboratory of Medical Molecular Biology, Institute of Basic Medical Sciences Chinese Academy of Medical Sciences, School of Basic Medicine Peking Union Medical College, Beijing, China; ^2^The Research Center for Medical Genomics, Key Laboratory of Medical Cell Biology, College of Basic Medical Science, China Medical University, Shenyang, China; ^3^Medical Genetics Institute, Henan Provincial People’s Hospital, People’s Hospital of Zhengzhou University, People’s Hospital of Henan University, Zhengzhou, China; ^4^Department of Pediatrics, the First Affiliated Hospital of Guangxi Medical University, Guangxi, China

**Keywords:** intellectual disability, triple T complex, TTI2, pathogenic mutations, whole-exome sequencing

## Abstract

Telomere maintenance 2 (TELO2)–interacting protein 2 (TTI2) interacts with TTI1 and TELO2 to form the Triple T complex, which is required for various cellular processes, including the double-strand DNA break response, nonsense-mediated mRNA decay, and telomerase assembly. Herein, we identified compound heterozygous mutations in *TTI2* using whole-exome sequencing (WES) in a Chinese family with a recessive inheritance pattern of syndromic intellectual disability. The patients displayed intellectual disability, aggressive and self-injurious behaviors, facial dysmorphic features, microcephaly, and skeletal anomalies. In addition, one patient showed cerebral white matter abnormality. Maternal novel indel mutation resulted in a premature termination codon and nonsense-mediated mRNA decay. Paternal reported c.1100C > T mutation changed the highly conserved proline to leucine that located in the DUF2454 domain. Immunoblotting experiments showed significantly decreased TTI2, TTI1, and TELO2 in the patients’ lymphocytes. These results indicated that *TTI2* loss-of-function mutations might cause an autosomal-recessive syndromic intellectual disability by affecting the Triple T complex. Our report expands the genetic causes of syndromic intellectual disability in the Chinese population.

## Introduction

Intellectual disability (ID) is a large group of neurodevelopmental disorders characterized by a congenital limitation in intellectual functioning (reasoning, learning, and problem solving) and adaptive behavior (conceptual, social, and practical skills), originating before the age of 18 years (American Psychiatric Association, 2013; Chiurazzi and Pirozzi, 2016). ID is estimated to affect 1% of the global population (American Psychiatric Association, 2013; Maulik et al., 2011). The prevalence of ID is 0.43% to 0.96% in China according to the 1987 and 2006 national sampling surveys of disability (Tian et al., 2017). ID is phenotypically heterogeneous and is frequently divided into two groups: syndromic ID, in which malformations in other organs or a typical (facial) *gestalt* are present, and nonsyndromic ID, in which no obvious comorbidities are present (Chiurazzi and Pirozzi, 2016).

Most IDs are considered to be caused by a complex mix of factors, including nongenetic and genetic factors (Yaqoob et al., 2004; Ropers, 2008; Ropers, 2010). Genetic factors account for 50% of ID cases, but an overproportionate fraction (possibly more than two-thirds) was observed in patients with moderate to severe ID (Shashi et al., 2014). The molecular mechanisms underlying ID are diverse, including large chromosomal abnormalities, submicroscopic copy number variants, and monogenic forms due to pathogenic variants in single genes (Kaufman et al., 2010; Piton et al., 2013; Jamra, 2018; Wieczorek, 2018). Potentially, more than 1,000 autosomal recessive ID genes exist; however, the vast majority remain unknown (Jamra, 2018). Due to the advent of next-generation sequencing, numerous candidate genes for ID have been identified (Najmabadi et al., 2011; Harripaul et al., 2017; Reuter et al., 2017).

*TTI2* (MIM#614426) maps on chromosomal 8p12 and has a genomic size of 40 kbs with eight coding exons. Full-length *TTI2* mRNA encodes telomere maintenance 2 (TELO2)–interacting protein 2 (TTI2), a regulator of the DNA damage response (DDR) localized both in the nucleus and cytoplasm (Genereaux et al., 2012). TTI2 interacts physically with TELO2-interacting protein 1 (TTI1) and TELO2 to form the evolutionarily conserved Triple T (TTT) complex. The TTT complex interacts with Hsp90 and the R2TP complex (RUVBL1, RUVBL2, RPAP3, and PIH1D1) forming a supercomplex to regulate the phosphatidylinositol 3-kinase–related kinase (PIKK) abundance and checkpoint signaling and is involved in various cellular processes, including DDR, nonsense-mediated decay, and telomerase assembly (You et al., 2016). To date, two missense *TTI2* mutations, c.1100C > T (p.Pro367Leu) and c.1307T > A (p.Ile436Asn), have been reported to cause nonsyndrome or syndromic ID in two unrelated consanguineous families originating from Iran and Algeria, respectively (Najmabadi et al., 2011; Langouët et al., 2013).

In the present study, we report the compound heterozygous mutations, c.942_944 delTCTinsCTGTGCTTCCATTCCTTCCTCCTAG (p.Leu315CysfsTer8) and c.1100C > T (p.Pro367Leu), in *TTI2* may be responsible for the syndromic ID phenotype in a nonconsanguineous family of Chinese origin.

## Materials and Methods

### Ethical Approval and Family History

**Figure 1:**
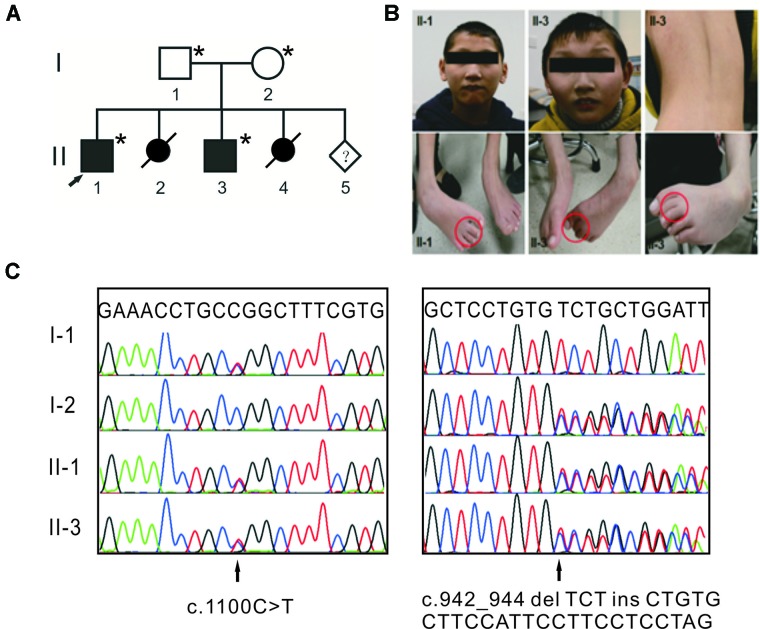
Compound heterozygous mutations in *TTI2* underlie syndromic intellectual disability (ID) in a Chinese family. **(A)** Pedigree of the indicated family. Squares and circles indicate males and females, respectively. Solid and open symbols denote the affected and unaffected individuals, respectively. Slashes indicate induced abortions, and an arrow indicates the proband. The individuals available for genotyping are denoted by asterisks. **(B)** Photographs of patients II-1 and II-3 showing thin lips, as well as strephenopodia and syndactyly phenotypes of the second and third toes. Individual II-3 showed dorsal lumbar scoliosis. **(C)** Sanger sequence chromatograms of affected individuals (II-1 and II-3) or heterozygous carriers (I-1 and I-2) with *TTI2* mutations. Letters over the chromatograms indicate the reference sequences. Black arrows indicate the site of mutations.

The study design was in accordance with the Helsinki Declaration and approved by the institutional review board of Peking Union Medical College. Written informed consent for the genetic analysis and the publication of this case report was obtained from the patients’ legal guardians. One family with syndromic ID was recruited from Henan province of China (**Figure 1A**). Two affected individuals were clinically evaluated, with particular attention to neurological, morphological, ophthalmological, and skeletal symptoms. Photographs of the face, trunk, and limbs were taken (**Figure 1B**). Their parents are healthy and have a nonconsanguineous marriage. The mother had two induced abortions.

### DNA Extraction and Quantification

Peripheral blood samples (3–5 ml) were collected from the affected individuals (II-1 and II-3) and their normal parents (I-1 and I-2). Genomic DNA was extracted from peripheral blood samples using the QIAamp DNA Blood Midi Kit (Qiagen, Hilden, Germany) and quantified using a Nanodrop 2000 spectrophotometer (Thermo Scientific, Waltham, MA, United States).

### Library Preparation, Emulsion PCR, and Whole-Exome Sequencing

The whole-exome library was prepared using the Ion AmpliSeq Exome RDY Library Preparation (Life Technologies, Carlsbad, CA, United States). Briefly, DNA amplification was conducted with 100 ng genomic DNA from the two affected individuals. The PCR reaction conditions included an initial 2-min denaturation step at 99°C followed by 10 cycles of denaturation (99°C) for 15 s, and annealing and extension (60°C) for 16 min. Sequencing adaptors that enabled sample multiplexing were ligated to the amplicons using the Ion Xpress Barcode Adapter (Life Technologies). The library was purified using AMPure XP Reagents (Beckman Coulter, Brea, CA, United States) following the manufacturer’s protocol. The Ion Library Quantitation Kit (Life Technologies) was used for quantification, and template-positive ion sphere particles (ISPs) were generated by emulsion PCR using the Ion One Touch 2 instrument (Life Technologies) according to the manufacturer’s protocol. ISPs were loaded and sequenced on an Ion Proton I chip using an Ion Proton machine.

### Data Analysis

Sequence data were aligned to the GRCh37/hg19 reference sequence with the Torrent Mapping Alignment Program. The Ion Torrent Variant Caller (version 4.4.3) was used for genotype calling of multiallelic substitutions and indels. The Integrative Genome Viewer (IGV, http://www.broadinstitute.org/igv/) and ANNOVAR (http://www.wannovar.usc.edu/) were used for sequence data visualization and variant annotation, respectively (Wang et al., 2010). To assess the pathogenicity of missense mutations, three types of prediction programs [SIFT, Polyphen2, and Rare exome variant ensemble learner (REVEL)] and two conservation programs (PhyloP and GERP++) were used. Effects on splicing were evaluated with Human Splicing Finder. The mutant protein stability was predicted with the online tools MUpro (http://mupro.proteomics.ics.uci.edu/) and I-Mutant v2.0 (http://folding.biofold.org/i-mutant/i-mutant2.0). ΔΔG < 0 means decreased stability, while ΔΔG > 0 indicates increased stability.

### Mutation Confirmation and Genotypic Mutation Assay

Sanger sequencing was performed to validate the identified mutations using WES in all available family members. Specific PCR and sequencing primers were designed using Primer Premier 5 (primer sequences are listed in **Table S1**). The *TTI2* indel or missense mutations were genotyped in DNA samples from 200 unrelated control subjects using either neutral polyacrylamide gel electrophoresis (PAGE) or restriction fragment length polymorphism–PCR with MspI, respectively.

### RNA Extraction, cDNA Synthesis, and Quantitative RT-PCR

**Figure 2:**
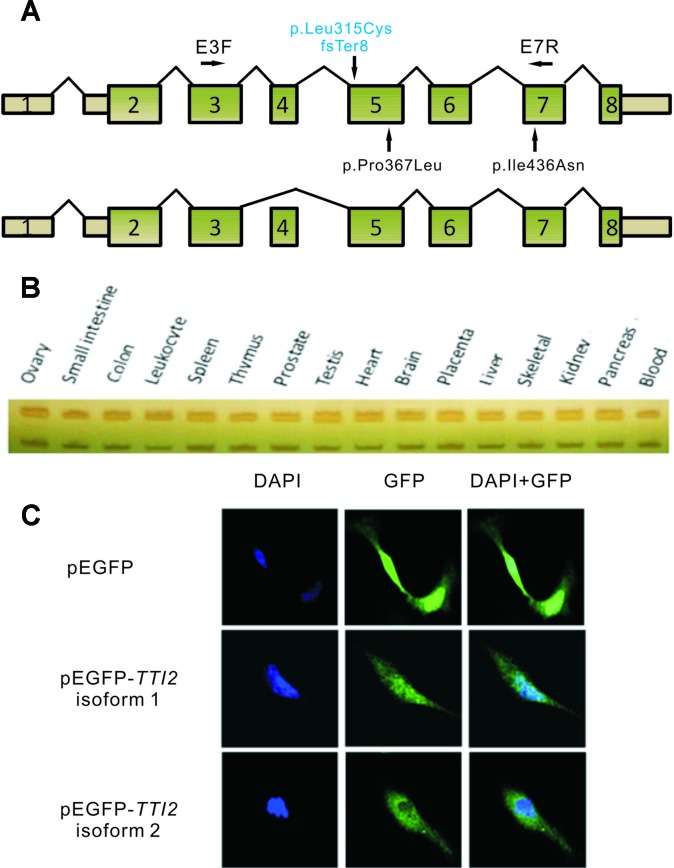
Schematic map and expression profile of two alternatively spliced *TTI2* isoforms. **(A)** Schematic map of *TTI2* showing the normal (upper) or variable spliced isoforms (lower), as well as the positions of mutations. Boxed regions denote exons, and connecting solid lines indicate introns. The mutation above the horizontal axis marked in blue is the novel frameshift mutation identified; those below are missense mutations reported to date. Primers E3F and E7R were used in RT-PCR. **(B)** Expression profile of the two *TTI2* isoforms in 16 types of normal human tissues. **(C)** SH-SY5Y cells were transfected with empty vector, GFP-tagged *TTI2* isoform 1 or isoform 2. Expressed GFP-TTI2 (green) and stained DAPI (blue) were observed.

Total RNA was extracted from peripheral blood leukocytes using Trizol LS (Invitrogen, Carlsbad, CA, United States), and 2 µg of RNA from each patient was subjected to reverse transcription using the GoScript^™^ Reverse Transcription System (Promega, Madison, WI, United States), according to the manufacturer’s protocol. RT-PCR was performed using primers E3F and E7R (NM_001102401.2, **Figure 2A**) with RNA from the two patients. The cDNA fragments produced were then sequenced using Sanger sequencing. To evaluate the amount of *TTI2* transcripts, quantitative RT-PCR (qRT-PCR) reactions were performed using primers TTI2-qPCR-F and TTI2-qPCR-R in a Roter-Gene 6000 instrument (Qiagen) with SYBR Premix Ex Taq (Takara Bio., Dalian, People’s Republic of China). Amplification conditions were 10 min at 95°C, followed by 40 cycles of 10 s at 95°C, 15 s at 60°C, and 20 s at 72°C. All reactions were run in quadruplicate. Glyceraldehyde 3-phosphate dehydrogenase was used as an endogenous control. Relative amounts of *TTI2* mRNA were calculated using the 2^–ΔΔCt^ method with the Roter-Gene Q series software (Livak and Schmittgen, 2001). The primer sequences are listed in **Table S1**. In addition, semiquantitative PCR was performed using primers E3F and E7R with cDNA from a human cDNA panel of 16 tissues (BD Biosciences Clontech, Palo Alto, CA), and the PCR productions were then separated by native PAGE.

### Construct Construction

A fragment was PCR-amplified with primers E3F and E7R from the parents’ cDNA and ligated into the pMD18-T vector (Takara Bio.) after purification. The vectors were subsequently sequenced using both forward and reverse primers. Wild-type *TTI2* was PCR-amplified from the parents’ cDNA, and two types of isoforms (1 and 2) were cloned into the expression vector pEGFP-C1 (BD Biosciences, Clontech) at the XhoI and HindIII restriction sites using T4 DNA ligase (New England Biolabs, Hitchin, United Kingdom). All constructs were verified using direct sequencing. The primer sequences are listed in **Table S1**.

### Cell Culture and Transfection

SH-SY5Y cells were grown on cover slips at 37°C and 5% CO_2_ in RPMI 1640 medium supplemented with 15% fetal bovine serum (Life Technologies) and 100 U/ml of penicillin/streptomycin. The recombinant pEGFP-C1 plasmid DNA (∼4 μg) was transiently transfected into cells using Lipofectamine 3000 reagent (Life Technologies), following the manufacturer’s instructions.

### Immunofluorescence Staining

Approximately 36 h after transfection, SH-SY5Y cells were washed once with phosphate-buffered saline (PBS) and fixed in 4% paraformaldehyde (4°C) for 20 min at room temperature. Cells were then washed in PBS and permeabilized in PBS plus 0.5% Triton X-100 for 30 min at room temperature. Nuclear staining was performed with DAPI. Confocal images were taken using a Zeiss LSM 510 META microscope (Oberkochen, Germany).

### Western Blotting

Immunoblot assays were performed on proteins extracted from lymphocytes obtained from individuals I-1, I-2, II-1, and II-3 using standard procedures. After denaturation at 99°C for 10 min, 30 µg of each protein sample was run on 10% sodium dodecyl sulfate–PAGE (PAGE) gels and then transferred to a polyvinylidene difluoride membrane (Millipore, Billerica, MA, United States). After the membranes were blocked with TBST (0.1% Tween-20 and 5% skimmed milk) for 1 h, they were incubated with primary anti-TTI2 antibody (1:1,500; Bethyl Laboratories, Montgomery, TX, United States; A303-476A), anti-TELO2 antibody (1:500; Abcam, Cambridge, UK; ab182525), and anti-TTI1 antibody (1:2,000; Abcam; ab176696) overnight at 4°C. The membranes were then incubated with horseradish peroxidase (HRP)–labeled secondary antibodies for 2 h. The HRP signal was detected using an enhanced chemiluminescent reagent kit (Millipore). Equal loading was confirmed by reprobing membranes with antibodies against β-actin (1:1,000; BioMed; BM0627). The signal intensities were analyzed with ImageJ software (Schneider et al., 2012).

## Results

### Clinical Characteristics of the ID Family

Clinical examination was performed on the two affected male siblings and summarized in **Table 1**. Both individuals with ID were born to their healthy nonconsanguineous parents with an unremarkable pregnancy and delivery (**Figure 1A**). Both patients had growth retardation and developmental delays in their infancy. Individual II-1 raised his head at 4 months, turned over at 10 months, walked at 4 years of age, and was wheelchair-dependent at 5 years; individual II-3 displayed similar symptoms but never walked independently. The two siblings were unable to use words, communicate with others, or feed themselves and displayed aggressive and self-injurious behaviors, with IQ below 35, which were tested at 8 months. The occipitofrontal head circumference of affected individuals varied between 2 and 6 standard deviation below the age-matched and sex-related means. Individual II-1 showed a sloping forehead, thin lips, anteverted large ears, strabismus, hypertonia, strephenopodia, and partial appearance of zygodactyly between the second and third toes (**Figure 1B**). Individual II-3 showed scoliosis and cerebral white matter abnormality in addition to sloping forehead, anteverted large ears, thin lips, hypertonia, and foot deformity (**Figure 1B**). They both have normal vision and hearing. Peripheral blood chromosome analysis was performed, and the karyotypes of individual II-3, his father, and mother were 46,XY, 46,XY, and 46,XX, respectively. Chromosomal microdeletions or microduplications (> 400 kb) were not observed in individual II-3 based on array-CGH analysis using the Agilent SurePrint G3 Human CGH Microarray kit.

**Table 1:** Clinical features of affected individuals with the compound heterozygous *TTI2* mutations in the indicated family.

Subject	II-1	II-3
Age (years)	17	12
Gender	Male	Male
Intellectual disability	+	+
Microcephaly	+	+
Cognitive impairment	+	+
Communication disorders	+	+
Irritability	+	+
Aggressive behavior	+	+
2/3 toe syndactyly	+	+
Strephenopodia	+	+
Scoliosis	-	+
Thin lips	+	+
Hypermyotonia	+	+
Movement disorders	+	+
Strabismus	+	-
Seizures	-	-
Hearing loss	-	-
Cortical visual impairment	-	-
Oral frenuli/ankyloglossia	-	-
Cleft palate	-	-
Congenital heart disease	-	-
Cerebral white matter abnormalities	NA	+

+, present; -, absent; NA, not available.

### Identification of Compound Heterozygous Mutations in TTI2

WES was performed on both affected individuals (II-1 and II-3) and the sequencing data analyzed based on autosomal recessive and X-linked inheritance pattern. Exome sequencing data were first filtered for variants in compound heterozygosity or homozygosity present in both affected siblings. Second, all variants with a minor allele frequency <0.01 [dbSNP150, the NHLBI ESP Exome Variant Server (EVS), the 1000 Genomes Project and Exome Aggregation Consortium (ExAC)] were retained, and we focused primarily on nonsense, missense, splice site mutations, and indels. Then, the variants were visually confirmed using IGV and further verified using Sanger sequencing. Only compound heterozygous mutations, c.942_944 delTCTinsCTGTGCTTCCATTCCTTCCTCCTAG (p.Leu315CysfsTer8) and c.1100C > T (p.Pro367Leu), in *TTI2* on chromosome 8p12 (referred to NM_025115) met our filtering criteria. Both mutations were in constitutively spliced exons. The father and mother were heterozygous for the p.Pro367Leu and p.Leu315CysfsTer8 mutations, respectively (**Figure 1C**). The missense mutation has been reported a pathogenic mutation with an allele frequency of 0.003% in the ExAC database, and the indel mutation was not present in the public databases. Both mutations were not detected in chromosomes from 200 ethnically matched control individuals. The p.Pro367Leu mutation altered a highly conserved residue in the DUF2454 domain and had SIFT, Polyphen2, and REVEL scores of 0.02 (damaging), 0.949 (probably damaging), and 0.621, respectively. The p.Leu315CysfsTer8 mutation may result in either synthesis of truncated protein products lacking the key DUF2454 domain of TTI2 or in degradation by nonsense-mediated mRNA decay (NMD). Filtering exome sequencing data based on X-linked inheritance pattern did not provide other candidate variants.

### Two Diverse TTI2 Isoforms

To investigate the effects of the indel or missense mutations on the mRNA transcripts of *TTI2*, RT-PCR assay was performed using primers E3F and E7R with RNA samples from the peripheral blood leukocytes of the two patients (**Figure 2A**). Sanger sequencing showed three overlapping peaks for each allele (data not shown). To test whether the mutations caused abnormal splicing of *TTI2*, the targeted fragments from the patients’ cDNA were amplified and then cloned into the pMD18-T vector for bacterial single colony sequencing. Sanger sequencing showed both mutations did not affect the normal splicing patterns. Interestingly, in addition to the full-length mRNA transcript (defined as isoform 1), which harbored the identified indel or missense mutations, a short mRNA transcript was found that lacked one exon compared with the full-length transcript (defined as isoform 2, **Figure 2A**). Subsequently, localization and expression profiles of the two *TTI2* isoforms were investigated. Expression analysis of both isoforms in 16 types of normal human tissues showed ubiquitous expression in all tested human tissues, such as heart, brain, and blood; however, isoform 1 was more abundant than isoform 2 (**Figure 2B**). Immunofluorescence staining showed both *TTI2* isoforms localized to the cytoplasm and nucleus in SH-SY5Y cells (**Figure 2C**).

### Expression of the Mutant TTI2

**Figure 3:**
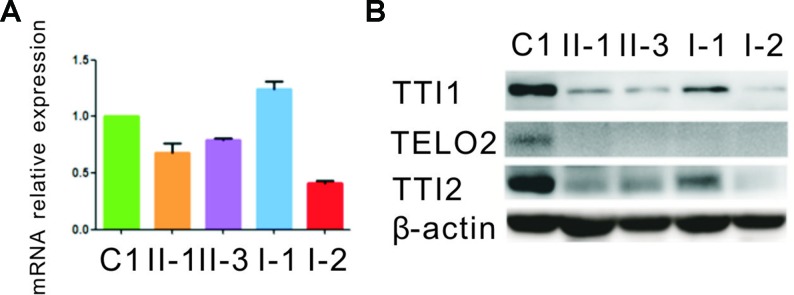
Quantitative mRNA and protein expression analysis of the *TTI2* mutations. **(A)** Quantitative RT-PCR (qRT-PCR) analysis of *TTI2* mRNA in peripheral blood leukocytes from patients (II-1 and II-3), unaffected parents (I-1 and I-2), and a control individual (C1). The results of the quantification of *TTI2* transcripts were first normalized to the *ACTB* housekeeping gene. The values presented are the means of triplicate determinations ± standard deviation (SD). The C1 was set to 1.0. **(B)** TTI1, TELO2, and TTI2 protein expression in lymphocytes obtained from the family members and C1, with β-actin serving as a loading control. The results represent one of three similar experiments.

qRT-PCR assay was performed to evaluate how the indel or missense mutation affected *TTI2* mRNA expression in peripheral blood leukocytes from the family members. An approximate 40% to 80% reduction of the *TTI2* mRNA level was detected in individuals I-2, II-1, and II-3, who harbored the indel mutation compared with a sample from a control individual (C1), indicating a partial degradation of the indel mutant transcripts. In addition, the mRNA expression was slightly upregulated in individuals I-1, II-1, and II-3, who possessed the missense mutation compared with C1 or I-2, who did not harbor the mutation, indicating a compensatory increase of the missense mutant transcript (**Figure 3A**). Subsequently, we used the online tools MUpro and I-Mutant v2.0 to predict the protein stability of the *TTI2* p.Pro367Leu mutation. Both of them suggested that the TTI2 p.Pro367Leu protein has an obviously decreased stability, with predicted ΔΔG as −0.41 and −1.11, respectively. Then, the effects of the indel or missense mutation on TTI2 protein expression were assessed in peripheral blood leukocytes from the family members using immunoblot assays. A decreased amount of TTI2 protein was found in peripheral blood leukocytes of the family members who possessed either one or two mutations compared with C1 (approximately 30%–65% of control levels, **Figure 3B**).

### Effects of the TTI2 Mutations on the Expression Level of TTI1 and TELO2

Next, the effects of the TTI2 mutations on the expression level of TTI1 and TELO2 in peripheral blood leukocytes were evaluated using immunoblot assays. The TTI1 levels were significantly decreased in all family members compared with C1 (approximately 17%–37% of control levels, **Figure 3B**), and TELO2 protein was almost undetectable in all family members compared with C1 (**Figure 3B**).

## Discussion

In this study, two deleterious compound heterozygous mutations, c.942_944delTCTinsCTGTGCTTCCATTCCTTCCTCCTAG (p.Leu315CysfsTer8) and c.1100C > T (p.Pro367Leu), were identified in *TTI2* using WES in a Chinese family with a recessive inheritance pattern of syndromic ID. Both mutations were not detectable in 200 controls and not present or present at a very low frequency (0.003%) in the public databases, indicating these mutations are not merely common silent polymorphisms. The qRT-PCR results showed the indel mutation led to a 50% degradation of the mutant transcript, indicating this mutation might exert a loss-of-function effect *via* an NMD mechanism. In addition, although the mRNA expression of the missense mutant transcript was slightly increased, the total TTI2 protein levels of the patients were significantly decreased compared with the controls; therefore, we speculate that TTI2 p.Pro367Leu destabilizes TTI2 protein through the ubiquitin–proteasome system and exerts a loss-of-function effect. Further immunoblot assays showed a significantly reduced amount of TTI1 and TELO2 proteins in the patients’ lymphocytes compared with the controls, verifying the previous results showing the mutations in *TTI2* impaired the stability of the TTT complex.

Previously, Najmabadi et al. identified homozygosity for a missense c.1100C > T (p. Pro367Leu) mutation in *TTI2* in four siblings with moderate nonsyndromic ID-39 (Najmabadi et al., 2011). Subsequently, Langouet et al. found that a missense c.1307T > A (p.Ile436N) mutation in *TTI2* causes a human autosomal recessive condition characterized by severe cognitive impairment, microcephaly, behavioral troubles, short stature, skeletal anomalies, and facial dysmorphic features (Langouët et al., 2013). To the best of our knowledge, this is the first report of mutations in *TTI2* in syndromic ID family of Chinese origin. The patients presented in this study also displayed growth retardation, ID, microcephaly, communication disorders, skeletal anomalies, and facial dysmorphic features. However, cerebral white matter abnormality (observed in individual II-3) has not been previously reported. Another component of the TTT complex is encoded by *TELO2*, which is mutated in families with the newly described You–Hoover–Fong syndrome characterized by ID, microcephaly and short stature, global developmental delay with no regression of learned skills, dysmorphic facial features, abnormal movements, and disturbed vision and hearing (You et al., 2016; Moosa et al., 2017). In addition, *TTI1*, a candidate pathogenic gene, was identified in a family with microcephaly and ID where a homozygous missense mutation (c.G2761A, p.D921N) segregated with the phenotype in two affected and four unaffected family members (Najmabadi et al., 2011). These reports, together with our results, further support the hypothesis of a crucial role of the TTT complex in brain development and functioning.

In addition to the full-length transcript of *TTI2*, a short mRNA transcript lacking exon 3 (isoform 2) was observed. Both transcripts were ubiquitously expressed in all tested human tissues, such as heart, brain, and blood; however, isoform 1 was more abundant than isoform 2. In addition, both isoforms localized to the cytoplasm and nucleus. However, the functional differences between the two isoforms require further study.

In previous studies, the TTT complex was shown to play an important role in the maturation and stabilization of the PIKKs (Takai et al., 2007; Hurov et al., 2010). You et al. found that significant reduction in the TTT complex due to the stress of cells exposed to 17AAG, an inhibitor of HSP90, led to reduced levels of at least two of the PIKKs (ATM, PRKDC) (You et al., 2016). Langouet et al. observed a similar decrease in protein levels of three PIKKs (ATM, DNA-PKcs, and MTOR) in the patients’ cells harboring the *TTI2* mutation compared with the controls (Langouët et al., 2013). In addition, patients with mutations in the TTT complex present with ID, delayed development, microcephaly, and dysmorphic features, which are common features of several PIKK-related disorders (Concannon and Gatti, 1997; Shanske et al., 1997; Baynam et al., 2015). The results from these studies indicate the PIKK signaling might be involved in the underlying molecular pathogenesis of syndromic ID. However, the exact mechanism remains to be elucidated. In addition, whether disruption of the TTT complex might have other functions than destabilization of the PIKKs requires further investigation.

In summary, our finding of the novel compound heterozygous *TTI2* mutations expands the genetic causes of ID in the Chinese population. Additionally, the results from this study combined with those of other investigators implicate that *TTI2* and the TTT complex play vital roles in human brain development.

## Ethics Statement

The study design was in accordance with the Helsinki Declaration and approved by the Institutional Review Board of Peking Union Medical College. Informed consent for the genetic analysis was obtained from the patients’ legal guardians.

## Author Contributions

RW, SH, HL, and XZ contributed to the conception and design of the study. RW, SH, HX, XY, AK, and JH contributed to the acquisition and analysis of data. RW, SH, and XZ contributed to the drafting of the manuscript and figures.

## Funding

This work was financially supported by the National Key Research and Development Program of China (grant no. 2016YFC0905100 and 2016YFC1000504), the CAMS Innovation Fund for Medical Sciences (CIFMS) (grant no. 2016-I2M-1-002), the National Natural Science Foundation of China (NSFC) (grant no. 81230015), the Beijing Municipal Science and Technology Commission (grant no. Z151100003915078), the Central Research Institutes of Basic Research and Public Service Special Operations (grant no. 2016ZX310161), the Medical Science and Technology Research Projects of Henan Provincial Health Bureau (grant no. 201601019), and the Scientific and Technological Projects of the Technology Bureau of Henan Provincial Technology (grant no. 172102410010).

## Conflict of Interest

The authors declare that the research was conducted in the absence of any commercial or financial relationships that could be construed as a potential conflict of interest.
