# Supplementary material for: Novel Compound Heterozygous Mutations in TTI2 Cause Syndromic Intellectual Disability in a Chinese Family
Source: Front Genet. 2019 Oct 29;10:1060. doi: 10.3389/fgene.2019.01060 (PMC6830114; doi:10.3389/fgene.2019.01060)
Supplement: Supplementary file 1 [file Table_1.docx]

Supplementary Material

# Supplementary Table S1 Primers used in the present study

| Name | Sequence (5’ to 3’) | Description |
| --- | --- | --- |
| TTI2-4F | TATGGACCCTAAGGACGG | Sense primer for validating the mutation of TTI2 |
| TTI2-4R | GAGTCAACTATGAGCACCTC | Antisense primer for validating the mutation of TTI2 |
| TTI2-control-F | GTCCTTCCATTCCTTCCTC | Sense primer for validating the mutation of TTI2 in control individuals |
| TTI2-control-missense-R | GCTGAGGTGGGAGGATTG | Antisense primer for validating the missense mutation of TTI2 in control individuals |
| TTI2-control-framshift-R | TGCGTAAAAGAAGGCGGT | Antisense primer for validating the framshift mutation of TTI2 in control individuals |
| E3F | ACTCTGCAACAGGTCACTCG | Sense primer for amplifying the cDNA fragment of TTI2 |
| E7R | CAAATCAGTTTCAAGAGGGC | Antisense primer for amplifying the cDNA fragment of TTI2 |
| TTI2-qPCR-F | TGAATGCGGTTCTGTGGC | Sense primer for the quantitative real-time PCR of TTI2 |
| TTI2-qPCR-R | ATGACCAATGATGCGGGA | Antisense primer for the quantitative real-time PCR of TTI2 |
| TTI2-EGFP-F | cctcgagggATGGAGCTTGACAGCGCTCTG | Sense primer for amplifying full length cDNA of TTI2 cloned into pEGFP-C1 vector |
| TTI2-EGFP-R | caagcttTTAAGTTCCATTGTAGGGTGCGCCT | Antisense primer for amplifying full length cDNA of TTI2 cloned into pEGFP-C1 vector |
| pMD18-M13F(-47)-seq | CGCCAGGGTTTTCCCAGTCACGAC | Sequences primer for identified insertion into pMD18 vector sequence |
| pEGFP-C-seq | CATGGTCCTGCTGGAGTTCGTG | Sequences primer for identified insertion into pEGFP-C1 vector sequence |

The capital letters denote TTI2-specific sequences, while the low-case letters indicate sequences containing restriction Site *XhoI* (ctcgag) or *HindIII*(aagctt)(underlined), which indicated in bold.
